# Emission color-tuned light-emitting diode microarrays of nonpolar In_x_Ga_1–x_N/GaN multishell nanotube heterostructures

**DOI:** 10.1038/srep18020

**Published:** 2015-12-09

**Authors:** Young Joon Hong, Chul-Ho Lee, Jinkyoung Yoo, Yong-Jin Kim, Junseok Jeong, Miyoung Kim, Gyu-Chul Yi

**Affiliations:** 1 Faculty of Nanotechnology & Advanced Materials Engineering, Graphene Research Institute, and Hybrid Materials Research Center, Sejong University, Seoul 143-747, Korea; 2 KU-KIST Graduate School of Converging Science and Technology, Korea University, Seoul 136-701, Korea; 3 Center for Integrated Nanotechnologies, Los Alamos National Laboratory, Los Alamos, NM 87545, United States; 4 Department of Physics & Astronomy and Institute of Applied Physics, Seoul National University, Seoul 151-747, Korea; 5 Department of Materials Science and Engineering, Research Institute of Advanced Materials (RIAM), Seoul National University, Seoul 151-744, Korea

## Abstract

Integration of nanostructure lighting source arrays with well-defined emission wavelengths is of great importance for optoelectronic integrated monolithic circuitry. We report on the fabrication and optical properties of GaN-based *p*–*n* junction multishell nanotube microarrays with composition-modulated nonpolar *m*-plane In_*x*_Ga_1–*x*_N/GaN multiple quantum wells (MQWs) integrated on *c*-sapphire or Si substrates. The emission wavelengths were controlled in the visible spectral range of green to violet by varying the indium mole fraction of the In_*x*_Ga_1–*x*_N MQWs in the range 0.13 ≤ *x* ≤ 0.36. Homogeneous emission from the entire area of the nanotube LED arrays was achieved via the formation of MQWs with uniform QW widths and composition by heteroepitaxy on the well-ordered nanotube arrays. Importantly, the wavelength-invariant electroluminescence emission was observed above a turn-on of 3.0 V because both the quantum-confinement Stark effect and band filling were suppressed due to the lack of spontaneous inherent electric field in the *m*-plane nanotube nonpolar MQWs. The method of fabricating the multishell nanotube LED microarrays with controlled emission colors has potential applications in monolithic nonpolar photonic and optoelectronic devices on commonly used *c*-sapphire and Si substrates.

Semiconductor nanostructures and heterostructures have attracted considerable attention for applications in future high-performance optoelectronics^1^,^2^, including nanolasers^3^,^4^ and optoelectronic integrated circuits^5^,^6^,^7^. In particular, one-dimensional nanostructures prepared by a bottom-up approach, such as nanorods, nanowires, and nanotubes, have important structural advantages compared with conventional planar films, including high-quality heteroepitaxial integration on (almost) arbitrary substrates (including III–V materials, Si, and glass) with a small footprint^8^,^9^,^10^; a high aspect ratio with a large active area^11^ for high photon extraction (or absorption) efficiency as a waveguide^12^; and structural versatility to designed three-dimensional heterostructures with compositional modulation in the axial^13^,^14^,^15^,^16^, radial^17^,^18^,^19^, and/or both directions^20^,^21^,^22^,^23^,^24^. These advantages have contributed to significant improvements in the efficiency of optoelectronic devices^2^,^12^,^14^,^25^.

GaN-based nanomaterials^26^ are promising for full-color lighting and optoelectronic device applications^20^,^25^,^27^,^28^,^29^,^30^,^31^ owing to the solid-phase solubility of the In_*x*_Ga_1–*x*_N alloy^30^,^32^,^33^. These nanostructures are typically grown along the polar *c*- or nonpolar *m*- or *a*- axes of wurtzite^34^,^35^,^36^. For this reason, the combination of such diverse growth directions with the axial^14^ or radial^25^ heterostructure geometries enables control over the polarity and optical properties of the heterostructures^15^,^37^. Recent developments of InGaN-based thin film light-emitting diodes (LEDs) have focused on semipolar or nonpolar heterostructures^38^,^39^ to resolve problems with the inherent internal electric field, which limits the efficiency of conventional *c*-plane planar LEDs^40^. Typically, *r*-and *m*-plane sapphire substrates are used to fabricate nonpolar *a*- and *m*-plane InGaN/GaN heterostructures, respectively^41^. The (100)*γ*-LiAlO_2_ substrate is often used to fabricate high-quality nonpolar InGaN-based LEDs, as this reduces the lattice mismatch to 1–2%^42^. Nevertheless, nonpolar InGaN heterostructures have an issue of the low indium incorporation for practical applications^38^. More importantly, the use of *c*-sapphire (or Si) is more cost-effective, and enables large-area growth. Thus, there is demand for the fabrication of nonpolar InGaN-based heterostructures on *c*-sapphire substrates with wide tunability of the indium composition. Such requirements for LED applications can be achieved by growing *c*-axis elongated nanostructures with radial nonpolar heterostructures^15^,^19^,^43^,^44^,^45^.

For the reasons, there have been intensive studies on GaN-based multishell heterostructures grown on *c*-axis nanowires. The catalyst-free, controlled growth of core GaN nanowires^36^,^46^,^47^ successfully produced regularly arrayed radial nanowire heterostructures with *m*-plane InGaN multiple quantum wells (MQWs)^21^,^48^ for realizing the uniform electroluminescence (EL) emission colors^19^,^43^,^44^. Meanwhile, a nanotube can be another candidate for the core template to grow the multishell heterostructure. Distinguished from the solid nanowires, there are several potential advantages to use the ZnO nanotube as a core template for fabricating multishell heterostructure LEDs rather. For example, the shape-controlled epitaxy of nanowalls offers more degrees of freedom in diversely designing the size, shape, and spacing of nanotube arrays^49^. This allows the fabrication of nano-LED arrays with the uniform EL emission. In addition, the metal-filled nanotube can further increase luminescent efficiency with surface plasmonic effect^50^ and/or efficient current injection through the metal core. Moreover, an ultrathin nanotube with a core cavity is expected to effectively release the strain caused by lattice misfit between GaN and ZnO through the large surface area^51^.

Here, we report on the fabrication and optical properties of GaN-based multishell nanotube heterostructure LED microarrays formed of composition-modulated *m*-plane {11–00} In_*x*_Ga_1–*x*_N/GaN radial MQWs grown on core ZnO nanotube arrays. The optical properties, including modulation of the emission wavelength of the nanotube LEDs, were investigated by measuring the complementary cathodoluminescence (CL), photoluminescence (PL), and EL. We further discuss these luminescent properties, and the correlation between the emission color and the spacing between nanotubes.

## Results and Discussion

### Structural and Compositional Characterizations of GaN Multishell Nanotube Heterostructures

**Figure 1:**
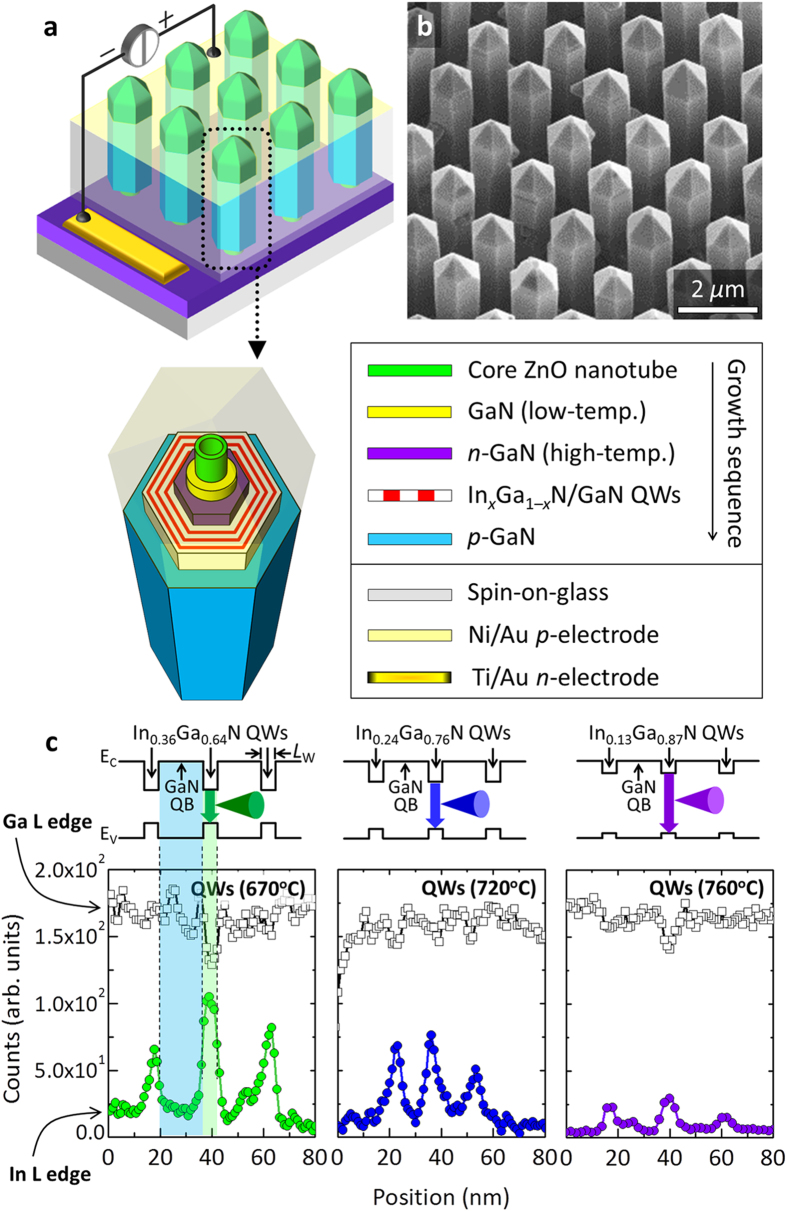
GaN-based multishell nanotube heterostructure LED arrays. (**a**) Illustration of the nanotube LED arrays and schematic diagram of a single nanotube LED. (**b**) Tilted view FESEM image of the nanotube LED arrays. (**c**) EDX intensity line profile of the L-characteristic rays of gallium (empty squares) and indium (solid circles) for nanotube MQWs grown at temperatures of 670, 720, and 760 °C (from left to right). The profiles were obtained along the radial direction from cross-section samples.

Multishell nanotube heterostructure LED arrays were fabricated on *n*^+^-GaN/Al_2_O_3_(0001) substrates using a patterned SiO_2_ growth mask via selective metal–organic vapor phase epitaxy (MOVPE). Figure 1a shows a schematic diagram of the vertical multishell nanotube heterostructures LED arrays, consisting of core ZnO nanotubes, GaN radial *p*–*n* junctions, and In_*x*_Ga_1–*x*_N/GaN MQWs. The core ZnO nanotube arrays had a mean height in the range 4.0−4.5 μm, and were prepared as a template for the growth of multishell nanotube heterostructures. The core diameter of the nanotubes was designed to be in the range 260−280 nm, and the core–core spacing (*i.e.*, pitch) was 2.5 μm; these features were defined using electron-beam lithographic patterning and subsequent etching of the SiO_2_ mask layer.

The multishell heterostructures were radially deposited on the outer surfaces of the ZnO nanotubes to form coaxial *p*–*n* junction LEDs. The GaN layer was coated on the ZnO nanotubes at a low growth temperature of 600 °C under nitrogen-ambient MOVPE growth conditions. Note that, without this low-temperature (LT) coating of the GaN layer (with a thickness of >50 nm), the core ZnO nanotubes were destroyed by the subsequent high-temperature process required for the MOVPE growth of the GaN-based LED structures (see Supplementary Fig. S1). The multishell layers of Si-doped *n*-GaN (with a thickness in the range 200–250 nm), three periods of In_*x*_Ga_1–*x*_N/GaN MQWs, and the outer shell of Mg-doped *p*-GaN (50–80-nm thick) were sequentially coated onto the LT-GaN/ZnO nanotubes using *in-situ* MOVPE. As a result of precisely-controlled selective-MOVPE growth process, the vertical nanotube LED arrays exhibited a well-defined spatial arrangement with uniform length and diameter, as shown by the field-emission scanning electron microscopy (FE-SEM) images in Fig. 1b. Details of the morphological changes and the microstructure of the radial multishell heterostructures are given in the Supplementary Information (see Figs S2–S4). Details of the fabrication and characterization can be found in the Methods section.

Controlling the indium mole fraction, *x*, in the In_*x*_Ga_1–*x*_N MQWs by adjusting the growth temperature provides a simple and systematic route to tuning the emission color of the nanotube LED arrays. We fabricated multishell nanotube MQW heterostructures with 2.5-nm-thick In_*x*_Ga_1–*x*_N layers and 20-nm-thick GaN layers at various growth temperatures in the range 670–760 °C using MOVPE. The In_*x*_Ga_1–*x*_N/GaN MQWs exhibited {11–00} (*i.e.*, *m*-plane) facets (see the transmission electron microscope (TEM) data shown in Fig. S3). Energy-dispersive x-ray (EDX) spectroscopy in scanning-TEM mode was used to determine the indium content in the In_*x*_Ga_1–*x*_N MQWs. Figure 1c shows compositional line profiles of the indium and gallium L edges measured along the radial direction of the In_*x*_Ga_1–*x*_N/GaN MQWs, revealing the formation of three pairs of MQWs. For the In_*x*_Ga_1–*x*_N MQWs grown at 670, 720, and 760 °C, the mean indium mole fraction was 0.36 ± 0.06, 0.24 ± 0.05, and 0.13 ± 0.03, respectively. These data were measured for a series of single nanotube LEDs; greater deviation of the indium content was observed in the In_*x*_Ga_1–*x*_N MQWs with a higher indium content, which was presumably due to the larger compositional fluctuations in the indium-rich growth conditions^52^,^53^.

### Luminescent Characteristics of Single GaN Multishell Nanotube Heterostructures for LED

The optical properties of single nanotube LEDs were characterized using CL spectroscopy. The CL from a nanotube LED (measured at 80 K) exhibited two dominant emission peaks centered at ~2.6 and 3.48 eV, which were attributed to emission from the InGaN MQWs (*I*^MQWs^) and donor-bound excitons in the core *n*-GaN (*I*^*n*-GaN^). The broad CL band in the range of 2.8–3.3 eV was attributed to band-to-Mg-doped acceptor emission and donor–acceptor pair recombination from the *p*-GaN outer shell layer (*I*^*p*-GaN^)^54^,^55^.

**Figure 2:**
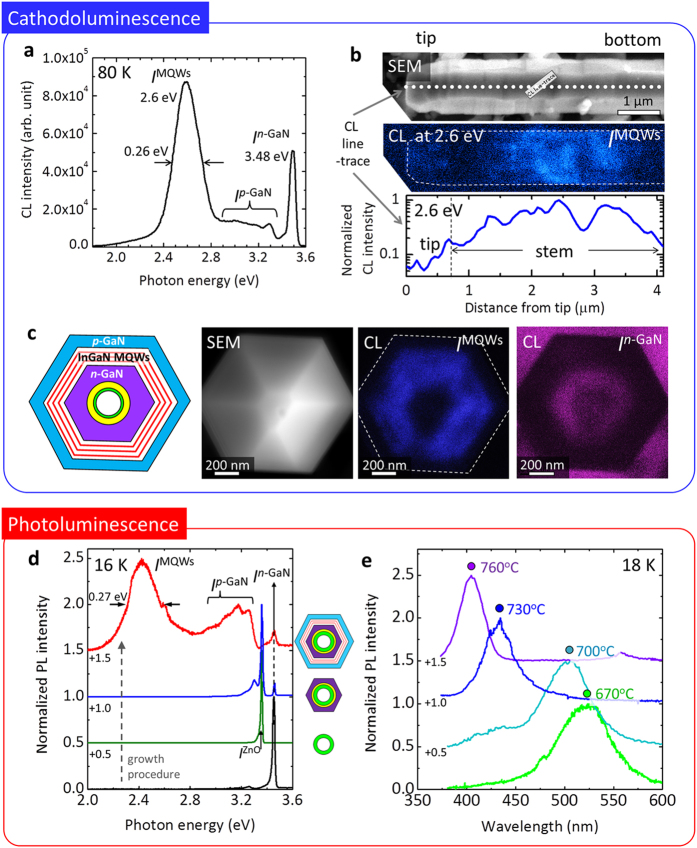
Optical characteristics of the multishell nanotube heterostructure. (**a**) CL spectra measured from single nanotube heterostructure at 80 K. (**b**) SEM image of stem of a nanotube LED and the corresponding monochromatic CL image mapped at a photon energy of 2.6 eV (upper panels); line profile of CL intensity for *I*^MQWs^ scanned along dotted line marked in the SEM image (bottom panel). (**c**) Top-view cross-sectional schematic, SEM image of single nanotube LED, and the corresponding monochromatic CL images mapped at photon energies of ~2.6 and 3.48 eV for *I*^MQWs^ and *I*^*n*-GaN^, from left to right, respectively. (**d**) PL specra of *n*-GaN substrate (black solid line), ZnO nanotube arrays (green), *n*-GaN/ZnO nanotube heterostructure arrays (blue), and multishell nanotube LED arrays (red) measured at 16 K. (**e**) PL spectra of multishell nanotube heterostructure arrays with In_*x*_Ga_1–*x*_N MQWs grown at temperatures of 670, 700, 730, and 760 °C.

Monochromatic mapping of the CL emission from a nanotube LED reveals spatial distribution of the *I*^MQWs^. Figure 2b shows an SEM image and corresponding monochromatic CL image scanned at a photon energy of 2.6 ± 0.04 eV for *I*^MQWs^ along the stem of a nanotube LED. Both the CL image and the line profile of the *I*^MQWs^ intensity revealed strong *I*^MQWs^ along the entire length of the nanotube sidewalls, suggesting that the In_*x*_Ga_1–*x*_N/GaN MQWs were formed conformally along the nanotube sidewalls.

Cross-sectional plan-view monochromatic CL mapping was used to visualize the spatial distribution of the distinct CL emissions. Figure 2c shows SEM images of single multishell nanotube heterostructures with a full LED structure, including the *p*–*n* junction and MQWs, together with a cross-sectional schematic diagram. From the monochromatic CL mapping at 2.6  ± 0.04 eV, *I*^MQWs^ was clearly observed from the MQW region sandwiched between the *n*-GaN core and the *p*-GaN shell. This indicates that *m*-plane InGaN MQWs with a hexagonal cylindrical geometry were successfully formed in the nanotube LED. In contrast, *I*^*n*-GaN^ was only observed from the core, which corresponds to *n*-GaN/LT-GaN; the strong *I*^*n*-GaN^ outside the nanotube was attributed to the *n*-GaN substrate.

The ensemble-averaged luminescent characteristics of the nanotube LED arrays were investigated using PL spectroscopy. Figure 2d shows a series of PL spectra measured at 16 K obtained from the samples following consecutive growth of an *n*-GaN thin film, ZnO nanotubes, an *n*-GaN coating, and a multishell coating of In_*x*_Ga_1–*x*_N/GaN MQWs and *p*-GaN. The PL spectra were measured from 8 × 10^3^ nanotube LEDs within the excitation laser spot. The strongest PL peak for the ZnO nanotubes was observed centered at 3.36 eV, which was attributed to neutral-donor-bound exciton emission (*I*^ZnO^)^56^. Importantly, we observed *I*^ZnO^ after forming the *n*-GaN layer, which indicates that the LT-GaN coating protected the core ZnO nanotubes from the high-temperature *n*-GaN deposition process in the ambient hydrogen environment (Supplementary Fig. S5). The PL of the nanotube LED arrays exhibited three dominant emission peaks, corresponding to *I*^*n*-GaN^ (3.46 eV), *I*^p-GaN^ (2.9–3.3 eV), and *I*^MQWs^ (2.4 eV) (see the red curve in Fig. 2d), similar to the CL spectra of a single nanotube LED. The full-width at half-maximum (FWHM) of *I*^MQWs^ was ~0.27 eV, which is broader than that of a typical thin film of In_*x*_Ga_1–*x*_N MQWs^57^ (*ca*. 0.12–0.15 eV). The broad spectrum was attributed to fluctuations in the indium content in the nanotube In_*x*_Ga_1–*x*_N/GaN MQWs, as observed using EDX spectroscopy. The FWHM of the *I*^MQWs^ CL was almost equal to that of the PL, which was presumably due to the homogeneous QW-luminescent characteristics over the entire area of the nanotube LED arrays.

The PL spectra of the nanotube MQWs were further investigated for the In_*x*_Ga_1–*x*_N/GaN (2.5/20-nm thick) MQWs grown at various growth temperatures of 670–760 °C. From the series of 16-K PL spectra shown in Fig. 2e, the PL peak position for *I*^MQWs^ gradually blueshifted from 525 nm (green) to 405 nm (violet) as the growth temperature increased from 670 to 760 °C. These results are consistent with the compositional analyses shown in Fig. 1.

### Electroluminescence of GaN Multishell Nanotube LEDs

**Figure 3:**
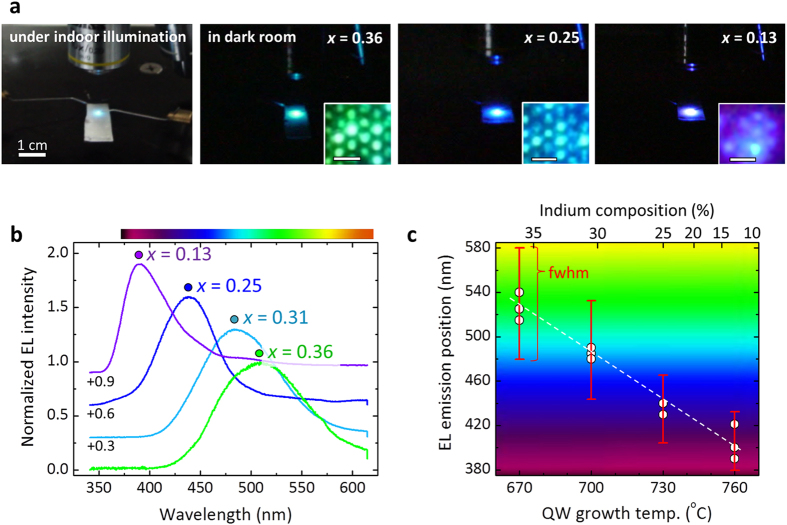
EL emission from multishell nanotube LED arrays. (**a**) Photographs of green, blue, and violet EL emissions from the multishell nanotube LED arrays with various indium contents in the InGaN MQWs. The insets show corresponding photomicrographs of visible EL emissions from the nanotube LED arrays. The scale bars of the insets are 5 μm. The LED chips in the photographs were operated at applied biases in the range 5–7 V. (**b**) EL spectra of the multishell nanotube LED arrays with In_*x*_Ga_1–*x*_N MQWs grown at temperatures of 670, 700, 730, and 760 °C. (**c**) Peak wavelength of the EL emissions plotted as a function of the QW growth temperature. The vertical red bars represent the FWHM of the EL peaks.

To inject current into the nanotube LED arrays, Ni/Au (10/10 nm) and Ti/Au (50/50 nm) Ohmic contacts were formed on the circumference of the outermost *p*-GaN shell and the *n*^+^-GaN substrate surface, respectively. A spin-on-glass (SOG) layer was used to provide electrical insulation between the two electrodes. Green, blue, and violet emissions from nanotube LED arrays with a chip size of 100 × 100 μm^2^ were observed at forward biases in the range 5–8 V, and were sufficiently strong to be observed with the naked eye under normal indoor illumination, as shown in Fig. 3a. Furthermore, EL from single-nanotube LEDs was clearly observed using an optical microscope, exhibiting green, blue, and violet emissions, depending on the indium content of the MQWs. The high brightness of these nanotube LEDs was attributed to the large internal quantum efficiency (IQE) of In_*x*_Ga_1–*x*_N (*x*~0.25 or ~0.31) nanotube MQWs, which was found to be 12 ± 4% by measuring the temperature-dependent PL spectra (Supplementary Fig. S6). This IQE is comparable to that of single-crystalline GaN nanowire-based LEDs^29^,^58^.

Figure 3b shows a series of room-temperature EL spectra of nanotube LED arrays with MQWs grown at temperatures of 670, 700, 730, and 760 °C. As with the PL spectra shown in Fig. 2e, the emission colors of the nanotube LED arrays were modulated from green to violet as the growth temperature increased. The EL peaks from the In_*x*_Ga_1–*x*_N MQWs with average mole fractions of 0.36, 0.31, 0.25, and 0.13 were centered at 510, 485, 440, and 390 nm, respectively. The FHWM of EL peaks gradually increased from 0.36 to 0.49 eV as the In mole fraction increased from *x* = 0.13 to 0.36 in the In_*x*_Ga_1–*x*_N MQWs (see Fig. 3b); this is consistent with the low-temperature PL spectra shown in Fig. 2e. In addition, the EL emission wavelength decreased monotonically as a function of the growth temperature from 510 nm (green) to 390 nm (violet), as shown in Fig. 3c. This was attributed to the temperature-dependent indium solubility of the *m*-plane radial MQWs formed in the nanotube geometry during MOVPE.

Dissimilar to other previous works, high indium content of *m*-plane sidewall InGaN QWs was achieved at relatively high growth temperature (Fig. 3c): *e*.*g*. Liao *et al.* reported *m*-plane InGaN QWs with indium mole fraction of 11–16% grown (with the nearest nanowire-to-nanowire spacing of 700 nm) at the growth temperature of 670–700 °C^48^, and Ra *et al.* demonstrated In_0.08_Ga_0.92_N coaxial QWs (with the spacing of ~200–300 nm) at 750 °C^19^. However, Koester *et al.* reported that the growth temperature of 750 °C yielded In_0.2_Ga_0.8_N MQWs (with the spacing of ~5–20 μm)^18^, implying that higher indium incorporation can be achieved with the greater nanowire spacing. It is well known that the lower density of nanostructure arrays enables more supply of precursors into the nanostructures during the growth, due to larger surface collection area^59^. This results in higher indium incorporation as observed in our nanotube LEDs with greater tube-to-tube spacing.

**Figure 4:**
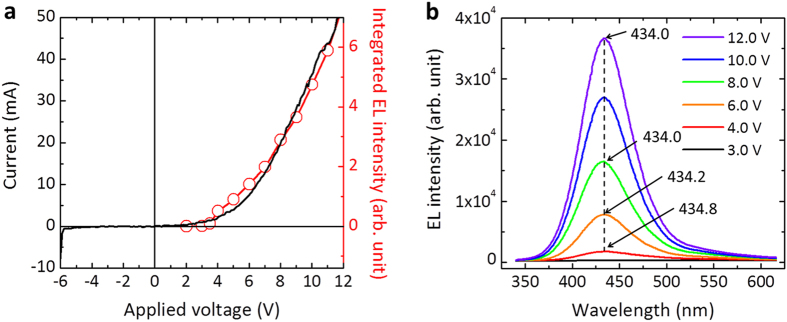
Electrical and EL characteristics of multishell nanotube LED arrays. (**a**) *I*–*V* characteristics (black solid curve) and EL intensity (red empty circles) as a function of the applied bias. (**b**) EL spectra of the nanotube LED arrays at various biases in the range 3.0–12.0 V.

Figure 4a shows the current–voltage (*I*–*V*) characteristics of the nanotube LED arrays, which exhibited the typical rectifying behavior of a *p*–*n* junction, with a threshold voltage of ~3–4 V, dynamic resistance of ~3 × 10^2^ Ω, and small reverse-bias leakage current of 3.5 × 10^−4^ A at an applied bias of –5 V. The turn-on bias for EL was ~3.5 V, which is similar to the electrical threshold voltage. The emission intensity increased rapidly with the applied bias voltage. A LED chip with 100 × 100 μm^2^ area is composed of a parallel circuit of 1.85 × 10^3^ nanotube LEDs, and more than 95% LEDs among them emitted the EL (see Supplementary Fig. S7). Accordingly, the dynamic resistance of a single nanotube LED was calculated to be ~5.6 × 10^5^ Ω, thus the electrical current flowing through each nanotube LED could be estimated to be a few μA at a typical operating forward electrical bias, which is consistent with previously reported values for single-nanowire LEDs^18^,^25^.

The EL emission exhibited very little blueshift of the peak position with increasing current, as shown in Fig. 4b. This was attributed to the absence of polarization-induced internal electric fields^60^ owing to the *m*-plane nonpolar nanotube MQWs, which is consistent with previous reports of single-nanowire LEDs^15^. We observed a small shift in the CL peak with increasing electron beam acceleration voltage in the range 5–15 kV (data not shown here), also indicating an absence of the quantum-confined Stark effect (QCSE) in the multishell MQWs. Such nonpolar features of the nanotube MQWs may be exploited to fabricate high-performance LEDs^40^ with suppressed QCSE^61^ or band-filling effects^62^. Applying a large bias did not result in additional EL peaks, such as emission from *p*-GaN due to electron overflow, which suggests that radiative recombination occurred exclusively in the nanotube MQWs.

Varying the geometrical arrangement represents another method to control the emission characteristics of nanotube MQWs since their growth rate is affected by the spacing between adjacent nanostructures. As the spacing increased from 2.5 to 6.0 μm, the EL wavelength shifted from 419 to 450 nm for a series of nanotube LED arrays that were prepared on a substrate during the same MOVPE growth batch (see Supplementary Fig. S8). This was attributed to an increase in the width *L*_w_ of the InGaN quantum wells of In_*x*_Ga_1–*x*_N MQW heterostructures with the larger nanotube spacing. It is well-known that increasing the nanostructure spacing leads to an increase in the surface collection area during growth, allowing more growth precursor to migrate to the nanostructures^63^. This results in a higher growth rate in the growth regime whereby the precursor is shared between nanostructures. Similarly, with our MOVPE growth, a larger nanotube spacing resulted in a faster growth rate (data not shown here); thus *L*_w_ increased with the nanotube spacing, leading to a longer wavelength of the emitted light. This observation shows that position-controlled selective growth of multishell nanotube LEDs enables homogeneous luminescence with control over the emission color. Further optimization of the MOVPE conditions is required to provide homogenous emission with narrow spectral linewidths over the entire range of visible wavelengths.

## Conclusion

We have demonstrated the fabrication of multishell nanotube LED arrays with composition-modulated nonpolar In_*x*_Ga_1–*x*_N/GaN MQWs using selective MOVPE. The emission color was tuned over the visible spectral range from green to violet by varying the indium content of the In_*x*_Ga_1–*x*_N MQWs, as confirmed by CL, PL, and EDX spectroscopic analyses. Homogeneous emission from the entire area of the nanotube LED arrays was achieved via the formation of MQWs with uniform *L*_w_ and *x* via heteroepitaxy on the well-ordered nanotube arrays. Importantly, both the QCSE and band filling were suppressed in the nanotube LED arrays. This method of fabricating LED microarrays with controlled emission colors has potential applications in monolithic nonpolar photonic and optoelectronic devices on commonly used *c*-sapphire and Si substrates.

## Methods

### Position-controlled selective MOVPE

The multishell nanotube heterostructure LED arrays were fabricated using selective MOVPE method. The core ZnO nanotube arrays were grown on *n*^+^-GaN/Al_2_O_3_(0001) substrates coated with hole-patterned SiO_2_ growth-mask layer (thickness of 50 nm). The hole-openings were designed to be a regular triangular arrangement with a typical hole diameter of 250 nm and pitch of 2.5 μm by using electron-beam lithography and wet chemical etching. Diethylzinc (DEZn) and high-purity oxygen (>99.9999%) were used as reactants with flow rates of 3.0 and 20 standard centimeters per minute (sccm), respectively, and high-purity argon as carrier gas. The reactor pressure and temperature were kept at 0.3 Torr and 600 °C for growing the ZnO nanotubes. The multishell heterostructures of LT-GaN layer (thickness of 60–80 nm), the multishell layers of Si-doped *n*-GaN (200–250 nm), InGaN/GaN (2.5/20 nm) MQWs, and outermost shell of Mg-doped *p*-GaN (50–80 nm), were sequentially coated onto the ZnO nanotubes using III–nitride MOVPE with vertical rotating type reactor. Trimethylindium, trimethylgallium and ammonia were employed as reactants for III–nitrides with hydrogen (or nitrogen) carrier gas. The growths of *n*- and *p*-GaN were performed at the substrate temperatures of 1080 and 1000 °C with doping precursors of ditertiarybutylsilane (flow rate of <0.3 sccm) and magnesocene (350 sccm), respectively. The typical resistivity and carrier concentration of the *n*-GaN were measured to be 6 × 10^–3^ Ω·cm and 4 × 10^18^ electrons cm^–3^, respectively; those of the *p*-GaN were 5–10 Ω·cm and 0.8–1.3 × 10^17^ holes cm^–3^ after *p*-type activation at 700 °C. In order to change the indium composition of In_*x*_Ga_1–*x*_N layers, MQWs-growth temperature was varied in the range of 650–800 °C.

### Device fabrication

The nanotube LEDs were fabricated by making Ohmic contacts of Ni/Au (10/10 nm) and Ti/Au (50/50 nm) bilayers on both the circumferential surface of outermost *p*-GaN shell and the *n*^+^-GaN, respectively. The two different metal electrodes were isolated with insulator by coating a spin-on-glass (SOG) layer between them. To expose the circumferential sidewall surface of nanotube heterostructures, wet chemical etching of SOG was performed using buffered-oxide etchant. The exposed sidewall height by etching was typically 1.5–2.0 μm (Supplementary Fig. S9a–c). To avoid the local current injection only through tips of individual nanotube LEDs, the Ni/Au electrode was conformally deposited on the sidewall surface of nanotube LEDs using an oblique angle metal evaporation technique (upper panel of Supplementary Fig. 9d). It is noted that the conformal deposition of continuous electrode layers on the circumference of nanotube heterostructures allows uniform current injection for operating entire nanotube LED arrays (see Supplementary Fig. S9d). Otherwise, the electrical current is not efficiently supplied into the nanotube LED arrays, which deteriorate the yield of EL emission. After the Ni/Au bilayer deposition, thermal annealing and sputtering deposition of indium tin oxide (150 nm) layer were performed for highly conducting semitransparent *p*-electrode.

### Characterizations

The morphology and structural characteristics were investigated using field-emission SEM (Philips, XL30SFEG) and high-resolution TEM (FEI, Tecnai G2 F20). For TEM analysis, samples were milled with cross sections orthogonal and parallel to the length direction by 30 kV-accelerated Ga ions using a focused ion beam machine (FIB; FEI, NOVA200 Nanolab) in the dual beam mode. The acceleration voltage of gallium ions was decreased from 30 to 5 kV at the finishing stage to reduce the damage of the sample and an inevitable contamination with gallium ions. The compositional line-profile of the In_*x*_Ga_1–*x*_N/GaN MQWs along its radial direction was obtained from EDX spectroscopy in the scanning-TEM mode of the TEM facility: the *x* was determined by measuring the relative ratio of x-ray counts of gallium and indium L edges.

The PL spectra were obtained at 16 K and room temperature using the 325 nm line of a continuous-wave He–Cd laser for excitation. A CL facility (Gatan, MonoCL3 + ) attached to the SEM (Hitachi, S-4300) was employed. The CL images and spectra were measured at 80 K and room temperature using a 10-kV electron beam. The spectral resolution of the employed high-resolution CL measurement system was as accurate as ± 8 meV. The measurements of EL spectra and *I*–*V* characteristics were performed simultaneously. The electrical and luminescent properties of the devices were performed by measuring *I*–*V* characteristic curves and EL spectra using sourcemeter (Keithley, 2600) and monochromator with charge-coupled device detector (Dongwoo Optron, MonoRa 150i), respectively. For the electrical measurement duty-cycle operation with a cycle period 20 ms and a pulse of 2 ms was used at room temperature.

## Additional Information

**How to cite this article**: Hong, Y. *et al.* Emission color-tuned light-emitting diode microarrays of nonpolar In_x_Ga_1-x_N/GaN multishell nanotube heterostructures. *Sci. Rep.* **5**, 18020; doi: 10.1038/srep18020 (2015).

## Supplementary Material

Supplementary Information
