# Supplementary material for: Emission color-tuned light-emitting diode microarrays of nonpolar InxGa1–xN/GaN multishell nanotube heterostructures
Source: Sci Rep. 2015 Dec 9;5:18020. doi: 10.1038/srep18020 (PMC4673456; doi:10.1038/srep18020)
Supplement: Supplementary Information [file srep18020-s1.doc]

Supplementary Information for

**Emission color-tuned light-emitting diode microarrays of nonpolar In_x_Ga_1–x_N/GaN multishell nanotube heterostructures**

Young Joon Hong^1,*^, Chul-Ho Lee^2,*^, Jinkyoung Yoo^3^, Yong-Jin Kim^4^, Junseok Jeong^1^, Miyoung Kim^5^, and Gyu-Chul Yi^4^

^1^Faculty of Nanotechnology & Advanced Materials Engineering, Graphene Research Institute, and Hybrid Materials Research Center, Sejong University, Seoul 143-747, Korea.

^2^KU-KIST Graduate School of Converging Science and Technology, Korea University, Seoul 136-701, Korea.

^3^Center for Integrated Nanotechnologies, Los Alamos National Laboratory, Los Alamos, NM 87545, United States.

^4^Department of Physics & Astronomy and Institute of Applied Physics, Seoul National University, Seoul 151-747, Korea.

^5^Department of Materials Science and Engineering, Research Institute of Advanced Materials (RIAM), Seoul National University, Seoul 151-744, Korea.

*These authors contributed equally to this work.

Correspondence and requests for materials should be addressed to Y.J.H. (email: [yjhong@sejong.ac.kr](mailto:yjhong@sejong.ac.kr)) and G.-C.Y. (email: [gcyi@snu.ac.kr](mailto:gcyi@snu.ac.kr))

**This file includes:**

Supplementary Figures and Captions 1–9;

Supplementary Descriptions;

Supplementary References

**Supplementary Figures and Descriptions**

**I. LT-GaN protection layer for growing multishell nanotube LEDs**


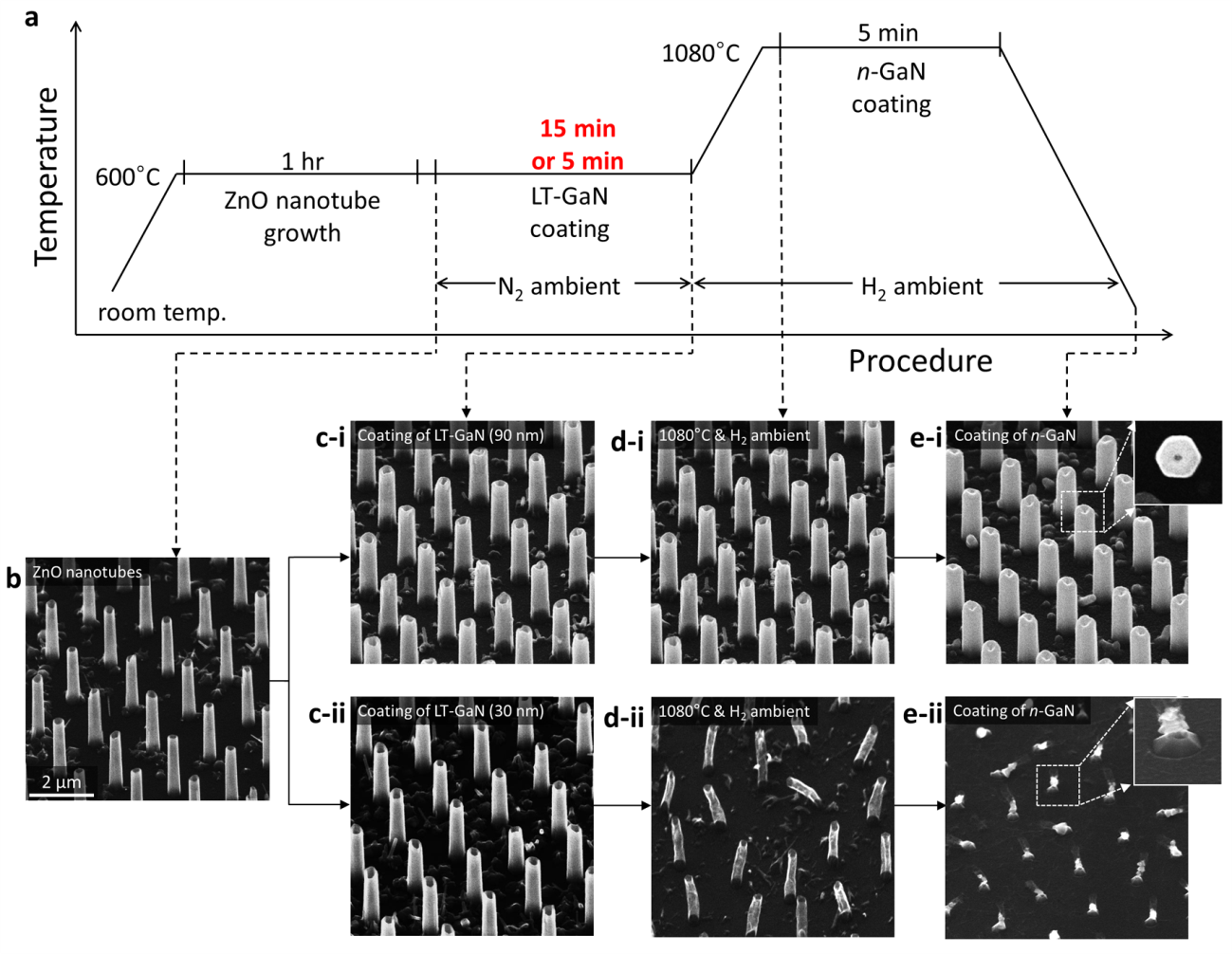


**Figure S1. LT-GaN protective layers for high temperature MOVPE process.** (a) Process diagram with temperature profile used for MOVPE growth of *n*-GaN/LT-GaN/ZnO nanotube heterostructres. Tilt-view SEM images of (b) ZnO nanotube arrays, (c) LT-GaN/ZnO nanotubes with LT-GaN thickness of (i) 90 nm and (ii) 30 nm, (d) the LT-GaN/ZnO nanotube arrays after ramping the temperature up to 1080°C under hydrogen ambient, and (e) the nanotube arrays after the coating process of Si-doped *n*-GaN layers on LT-GaN/ZnO nanotubes.

It is well known that high quality GaN crystals are grown at high growth temperature in the range of 900–1100°C for MOVPE process. However, the ZnO nanotubes have no tolerance against such high temperature with hydrogen ambient. Hence, we employed low-temperature coating process of GaN for the following high-temperature growth of multishell heterostructure of GaN *p*–*n* junction and InGaN MQWs. Figure S1a depicts a growth process diagram with temperature profile. After the growth of ZnO nanotube arrays (Fig. S1b), the heteroepitaxial coating of GaN was performed at low growth temperature of 600°C with different growth time of 5 and 15 min, resulting in LT-GaN thickness of ~30 and 90 nm, respectively (Fig. S1c). Sequentially, Si-doped *n*-GaN was homoepitaxially coated on these GaN/ZnO nanotube heterostructures at typical GaN growth temperature of 1000–1080°C, as depicted in Fig. S1a.

Figure S1d represents that the LT-GaN (90 nm)/ZnO nanotubes kept their general morphologies from high temperature process, whereas the LT-GaN (30 nm)/ZnO nanotubes were seriously damaged and degraded under the same process conditions. This result suggested the sufficiently thick LT-GaN layer is required for heteroepitaxial coating of multishell heterostructures. It is noted that at least 50 nm-thick LT-GaN protection layer is necessary for the tolerance at high temperature process above 1000°C. It is also noted that the high-temperature growth of *n*-GaN yielded hexagonally facetted prismatic structure with six *m*-plane sidewalls (Fig. S1e) for formation of *m*-plane nanotube MQWs.

**
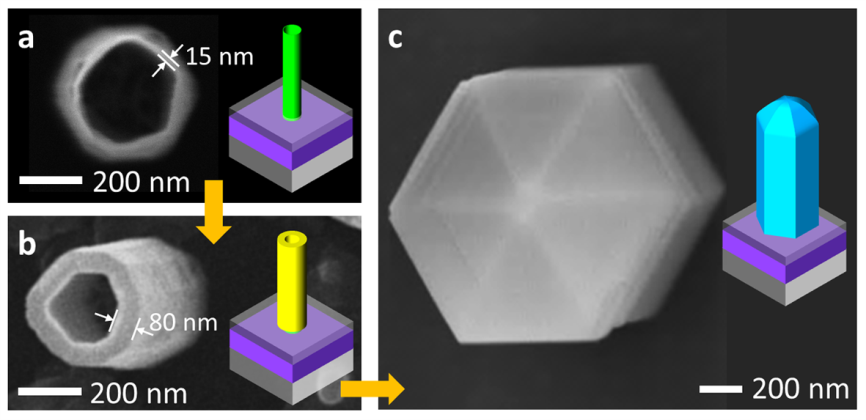
**

**Figure S2. Top-view FESEM images.** (a) core ZnO nanotube, (b) LT-GaN-coated ZnO nanotube, and (c) multishell nanotube heterostructure LED.

FigureS2 is a series of top-view FESEM images of (i) ZnO nanotubes, (ii) GaN/ZnO nanotube heterostructures, and (iii) multishell nanotube heterostructure LEDs, demonstrating the radial deposition of multishell heterostructures. The wall thickness of the nanotube was conformally increased from 15 to 80 nm by the radial growth of the LT-GaN protective layer on the ZnO nanotube (Fig. S2a–b). After the MOVPE of the LED structure, the multishell nanotube heterostructures exhibited regular hexagonal prismatic morphology with typical diameter of ~1.2 μm (Fig. S2c), implying the single crystalline nature of the multishell nanotube heterostructures. As a result, the height of nanotubes did not change significantly during the growth, whereas the nanotube diameter increased, indicating the preferential growth of LED structures along the radial direction.

**II. TEM observation of nanotube cross section orthogonal and parellel to legnth direction**


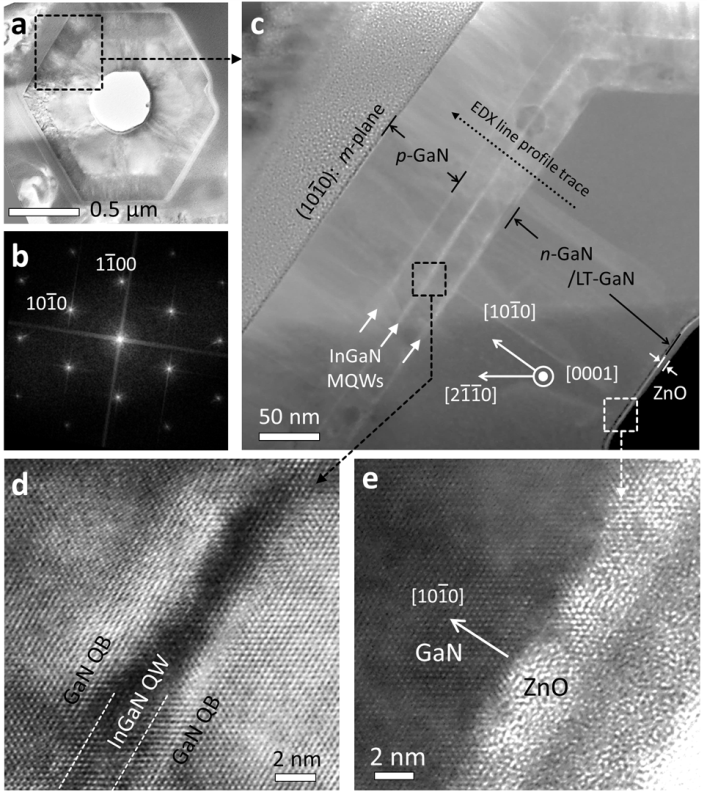


**Figure S3. Horizontal cross-sectional TEM observation of multishell nanotube heterostructure.** (a) Low magnification scanning-TEM HAADF image. (b) Corresponding diffraction patterns obtained through FFT process. (c) High magnification scanning-TEM HAADF image. High-resolution TEM images recorded around (d) InGaN/GaN QW and (e) core LT-GaN/ZnO regions.

The formation of single crystalline multishell heterostructures of the GaN *p*–*n* junction and the InGaN/GaN MQWs was investigated using TEM. The cross-sectional specimens orthogonal to the tube length direction were prepared. Low-magnification TEM image of Fig. S3a clearly displays the multishell tube geometry with a cylindrical core cavity and well-facetted six sidewalls. Corresponding diffraction patterns, obtained through fast Fourier transform (FFT) of the TEM image, demonstrated that the multishell heterostructures were grown to be wurtzite single crystalline with six {100} (*m*-plane) sidewall facets (Fig. S3b). In the high-magnification, high-angle annular dark-field (HAADF) scanning-TEM image of Fig. S3c, the multishell layers of a core ZnO tube, *n*-GaN/LT-GaN, three-period InGaN/GaN MQWs (three lines with bright contrast correspond to the InGaN MQW layers) and the outermost *p*-GaN shell were clearly observed with sharp interfaces. These InGaN/GaN MQWs were found to form non-polar *m*-plane (100) superlattices with QW width (*L*_w_) of 2.4–2.8 nm and quantum barrier (QB) thickness of 18–25nm (Fig. S3d). The formation of single crystalline multishell LED structures is attributed to the heteroepitaxy of high-quality LT-GaN on ZnO, shown in Fig. S3e.

**
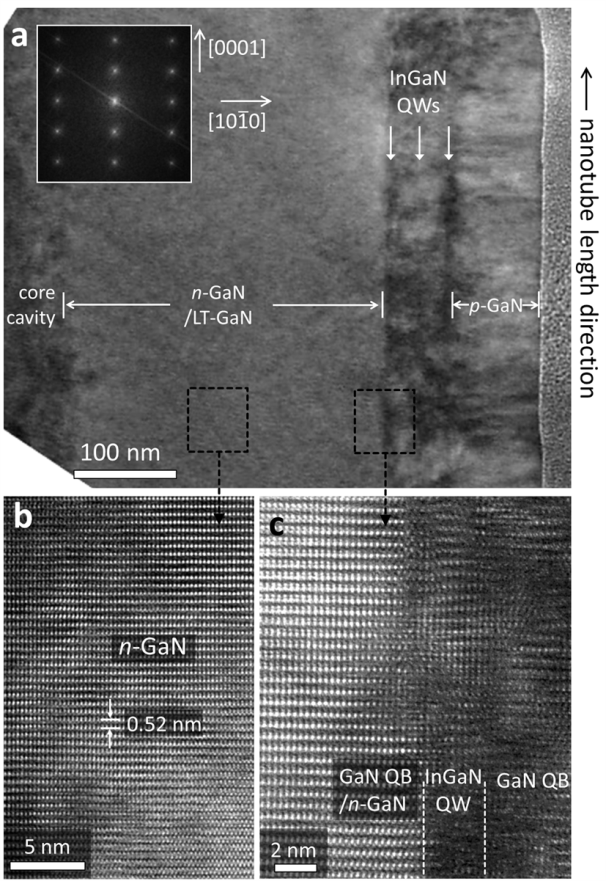
**

**Figure S4. Vertical cross-sectional high-resolution TEM observation**. (a) Low magnification TEM image showing the multishell heterostructures including GaN *p*–*n* junction with MQWs. Lattice images of (b) *n*-GaN and (c) InGaN/GaN QW regions. The inset in (a) is corresponding diffraction patterns obtained through FFT process. The images are taken along <20>.

The vertical cross-sectional TEM observation, taken along the <20> zone axis, also confirmed the formation of multishell nanotube heterostructures (Fig. S4a). The lattice image of Fig. S4b presents single crystallinity of *n*-GaN: extended crystal defects of stacking faults or dislocations were rarely observed^S1^, presumably owing to small lattice misfit of 0.4% along *c*-axis (*c*_GaN_=5.185 Å & *c*_ZnO_=5.204 Å). However, in contrast to the vertical cross sectional inspection, some of misfit dislocations are found preferentially along the [100] in the cross section normal to length direction, presumably due to the in-plane lattice mismatch of 1.9% (*a*_GaN_=3.189 Å & *a*_ZnO_=3.250 Å). The size-dependent formation of the dislocations in the tubular structure was further discussed elsewhere^S2^. The lattice image of InGaN/GaN MQWs (Fig. S4c) presents the QW *L*_w_ of ~2.5 nm closely matched to the horizontal cross-sectional observation. The TEM analyses demonstrated all the multishell heterostructures were formed to be single crystalline with well-defined heterointerfaces, indicating high-quality nanotube LED structures.

**III. Low-temperature PL spectra of multishell nanotube heterostructures**


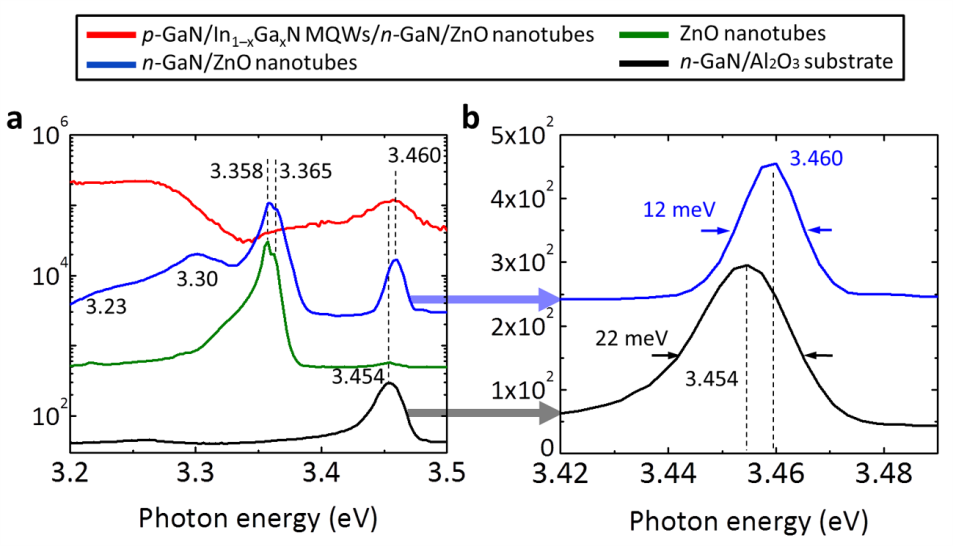


**Figure S5. Optical properties of the multishell nanotube heterostructure arrays.** (a) High-resolution PL spectra plotted with logarithmic scale, measured in the photon energy range of 3.2–3.5 eV. (b) High-resolution PL spectra measured in the vicinity of NBE of *n*-GaN substrate (black) and *n*-GaN/ZnO nanotube heterostructure arrays (blue).

For the ZnO nanotubes, the strongest peak of *I*^ZnO^ was clearly resolved into two distinct peaks at 3.358 and 3.365 eV (green solid line, Fig. S5a), ascribed to neutral-donor-bound exciton peaks (*I*_2_). Importantly, these *I*_2_ peaks were still observed to be resolved after the coating of *n*-GaN (blue line) presumably because the LT-GaN coating well protected the core ZnO nanotubes from high-temperature process of *n*-GaN coating (1080°C). Other PL peaks at 3.30 and 3.23 eV in *n*-GaN/ZnO nanotubes (blue line, Fig. S5a) are tentatively assigned to donor–acceptor pair recombination and first-order longitudinal optical phonon replica of core ZnO, respectively^S3^.

In particular, as shown in Fig. S5b, the *I^n^*^-GaN^ from nanotube *n*-GaN (blue line) exhibited much narrower fwhm (12 meV) of PL peak than that of *n*-GaN seed substrate (22 eV) formed on *c*-Al_2_O_3_ (black), also the *I^n^*^-GaN^ peak position of *n*-GaN shell layer was 3.460 eV rather than 3.454 eV of PL peak of *n*-GaN substrate. This observation suggested high crystal and optical quality^S4^ of the *n*-GaN shell layer rather than those of *n*-GaN substrate, presumably owing to sufficient relaxation of residual in-plane strain through the large surface area of nanotube sidewalls, which may be beneficial for fabricating high quality non-polar LED structures.

**IV. Temperature-dependent PL of InGaN MQWs grown on nanotubes**

***
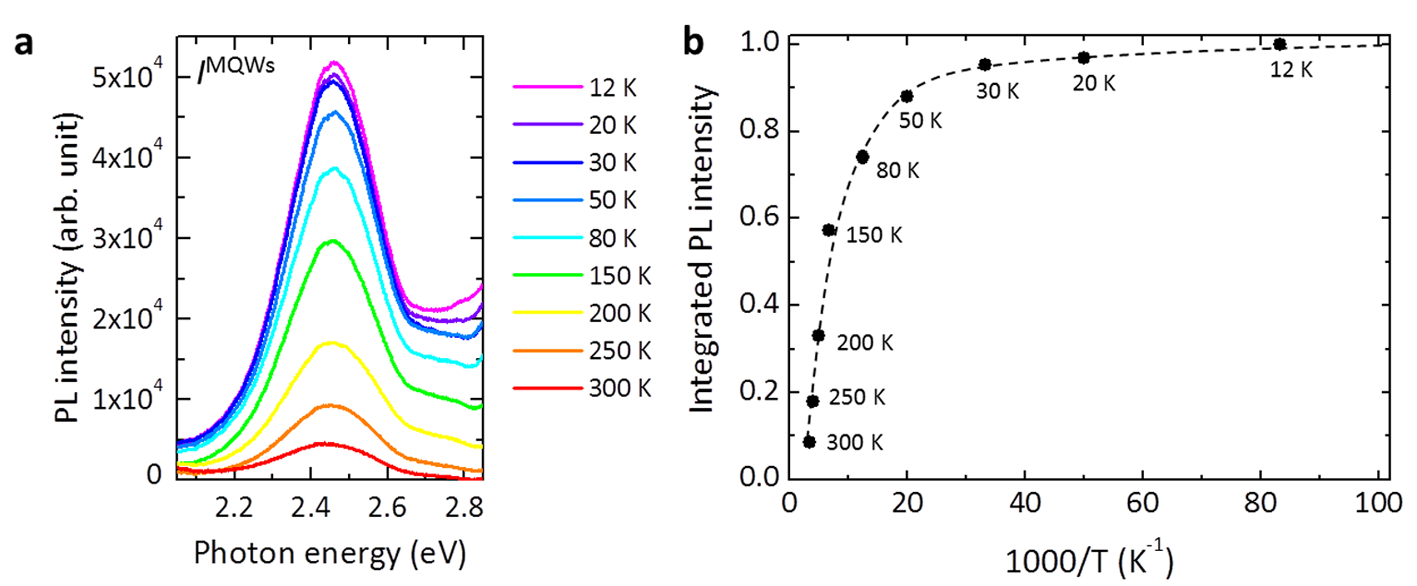
***

**Figure S6.** (a) Temperature-dependent PL spectra of In*_x_*Ga_1–_*_x_*N/GaN MQWs (*x*~0.31) grown on nanotubes. (b) PL intensity as a function of reciprocal temperature for the InGaN MQWs.

**V. EL emission from nanotube LED arrays**


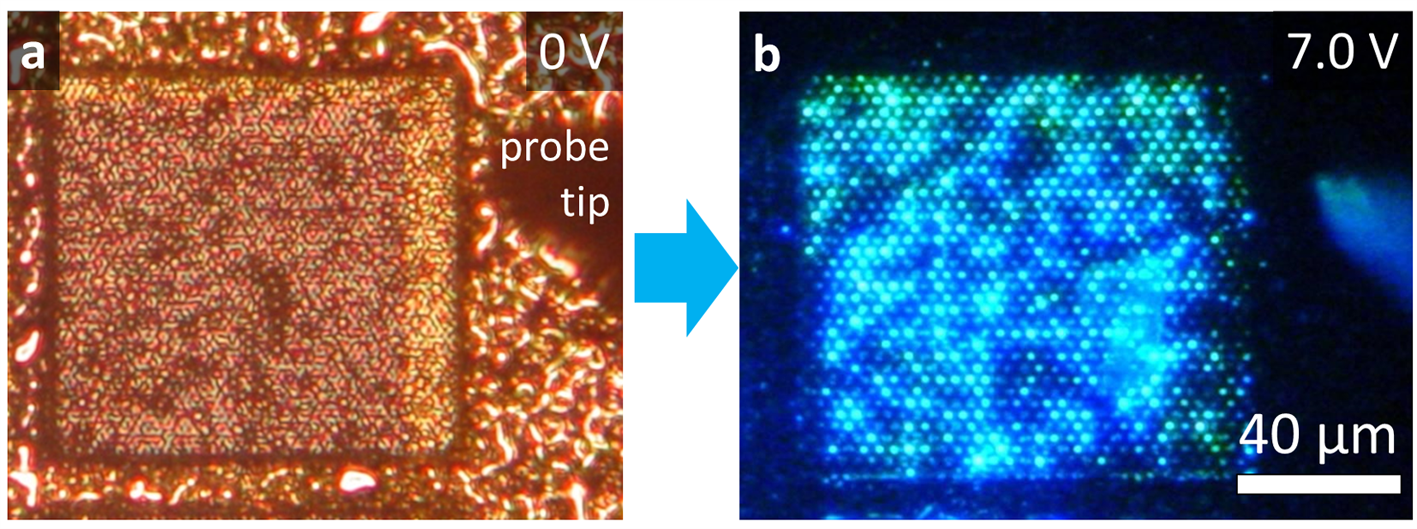


**Figure S7. Photomicrograph of a LED chip** (a) at no applied bias voltage under normal optical microscopic illumination condition and (b) operating at applied voltage of 7.0 V in a dark room.

**VI. Correlation of nanotube LED spacing and EL peak position**


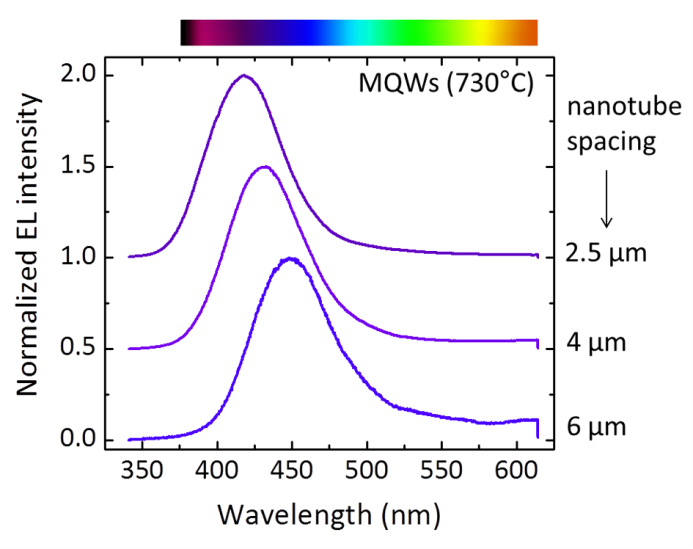


**Figure S8. EL spectra of nanotube LEDs with different nanotube spacings of 2.5, 4, and 6 μm fabricated on a substrate in the same MOVPE batch (MQW growth tempeature of730°C).**

**VII. Device fabrication processing & Top electrode metalization for high EL emission yield of nanotube LED arrays**


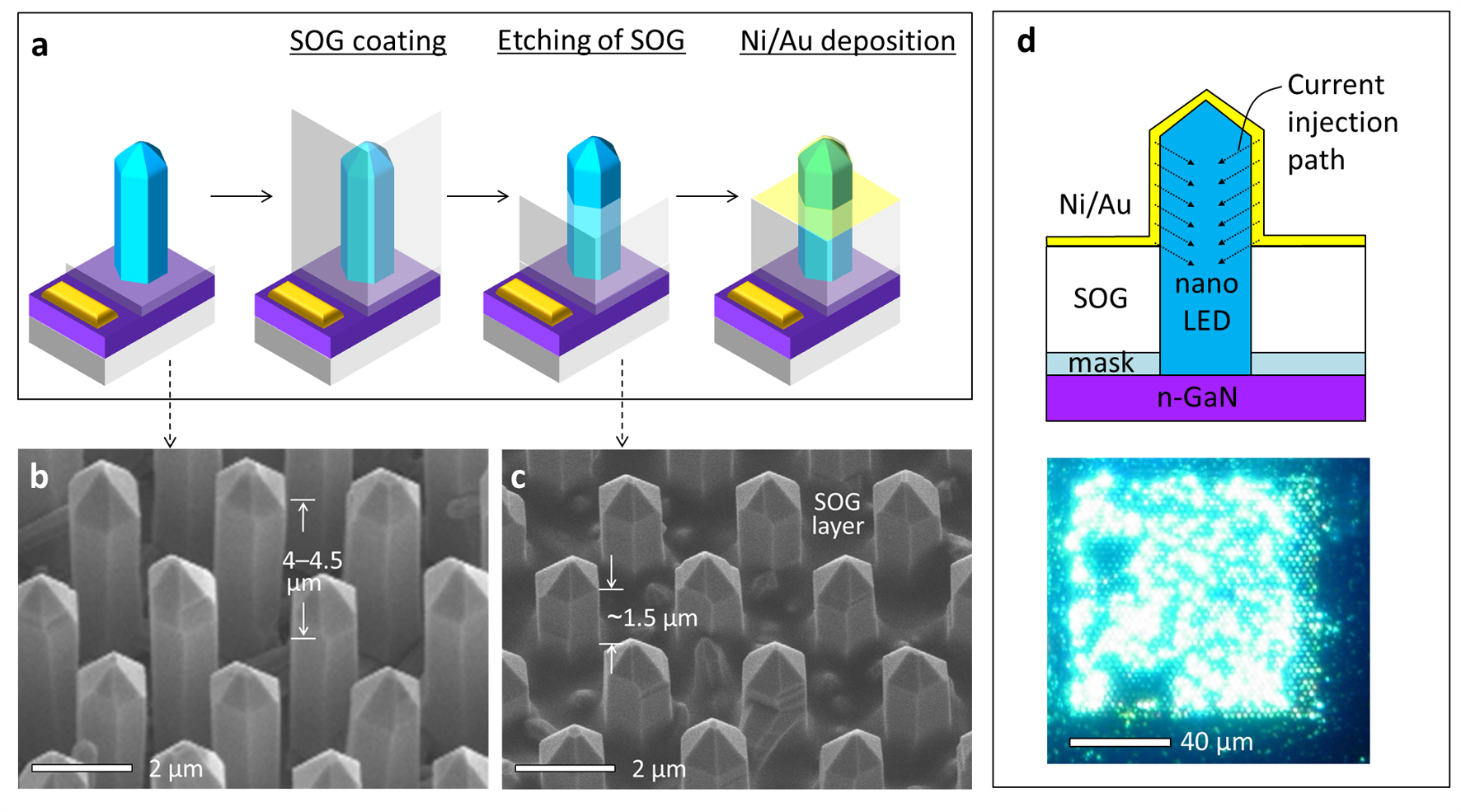


**Figure S9.** (a) Schematic illustration of device fabrication processing. Tilt-view SEM images of nanotube LED arrays (b) before coating of the insulating SOG layer and (c) after etching of the SOG layer to expose the surface of nanotube LED sidewalls for conformal deposition of the Ni/Au electrode on the exposed sidewalls. (d) Cross-sectional schematic of the Ni/Au top electrode coated on a nanotube LED (upper panel) and the EL emission photomicrograph of a 100×100 μm^2^ chip (lower panel).

**Supplementary References**

S1. Hong, Y.J. et al. Structural and optical characteristics of GaN/ZnO coaxial nanotube heterostructure arrays for light-emitting device applications. *New J. Phys.* **11**, 125021 (2009).

S2. Yoon, A. et al. Geometry-Induced Dislocations in Coaxial Heterostructural Nanotubes. *Small* **9**, 2255–2259 (2013).

S3. Meyer, B.K. et al. Bound exciton and donor–acceptor pair recombinations in ZnO. *Phys. Status Solidi B* **241**, 231–260 (2004).

S4. Cremades, A., Görgens, L., Ambacher, O., Stutzmann, M. & Scholz, F. Structural and optical properties of Si-doped GaN. *Phys. Rev. B* **61**, 2812–2818 (2000).
